# An updated LSU database and pipeline for environmental DNA identification of arbuscular mycorrhizal fungi

**DOI:** 10.1007/s00572-024-01159-3

**Published:** 2024-06-29

**Authors:** Camille S. Delavaux, Robert J. Ramos, Sidney L. Stürmer, James D. Bever

**Affiliations:** 1 Institute of Integrative Biology, ETH Zurich (Swiss Federal Institute of Technology), Universitätsstrasse 16, Zurich, 8092 Switzerland; 2 The Environmental Data Science Innovation & Inclusion Lab (ESIIL), University of Colorado Boulder, Colorado, 80309 USA; 3 Departamento de Ciências Naturais, Universidade Regional de Blumenau, Blumenau, Santa Catarina 89030-903 Brazil; 4 Department of Ecology and Evolutionary Biology, The University of Kansas, 2041 Haworth Hall, 1200 Sunnyside Avenue, Lawrence, KS 66045 USA; 5 Kansas Biological Survey, The University of Kansas, 106 Higuchi Hall, 2101 Constant Avenue, Lawrence, KS 66047 USA

**Keywords:** Large subunit rDNA, Phylogenetics, Arbuscular mycorrhizal fungi, Environmental DNA sequences, Illumina sequencing

## Abstract

Recent work established a backbone reference tree and phylogenetic placement pipeline for identification of arbuscular mycorrhizal fungal (AMF) large subunit (LSU) rDNA environmental sequences. Our previously published pipeline allowed any environmental sequence to be identified as putative AMF or within one of the major families. Despite this contribution, difficulties in implementation of the pipeline remain. Here, we present an updated database and pipeline with (1) an expanded backbone tree to include four newly described genera and (2) several changes to improve ease and consistency of implementation. In particular, packages required for the pipeline are now installed as a single folder (conda environment) and the pipeline has been tested across three university computing clusters. This updated backbone tree and pipeline will enable broadened adoption by the community, advancing our understanding of these ubiquitous and ecologically important fungi.

**Supplementary Information:**

The online version contains supplementary material available at 10.1007/s00572-024-01159-3.

## Introduction

The use of environmental DNA and amplicon sequencing has become a fundamental tool for fungal ecologists (Tedersoo et al. 2022), and particularly for those studying mycorrhizal fungi. These approaches enable researchers to obtain a relatively unbiased snapshot of the community using a very small amount of starting material (e.g., soil or roots). In theory, this should generate large amounts of data to understand the biogeography and ecology of this important group of fungi. However, the most common type of mycorrhizal fungi – the arbuscular mycorrhizal fungi (AMF, phylum Glomeromycota) – are not well represented when using the “universal” fungal primers targeting the internal transcribed spacer of the rDNA gene (Schoch et al. 2012; Lekberg et al. 2018; Tedersoo et al. 2022). Furthermore, AMF sequence databases are geographically biased and incomplete (Bidartondo et al., Öpik et al. 2010; Stockinger et al. 2010). Therefore, the full potential of this technology for AMF requires use of other gene regions and phylogenetic placement approaches.

Although there is no consensus on the ideal region to target or sequencing platforms to use for AMF, the large subunit (LSU) amplicon (short read) sequencing is a good choice for next generation sequencing (Illumina; Stockinger et al. 2010; Delavaux et al. 2021, Delavaux et al. 2022). This region allows phylogenetic placement and subsequent discovery of novel taxa, while showing good taxonomic resolution at the species level (Krüger et al. 2009; Hart et al. 2015; House et al. 2016). Previous work established a curated reference tree and developed a pipeline for phylogenetic placement of AMF for the LSU region (Delavaux et al. 2021, 2022). Specifically, this work enabled phylogenetic placement of any environmental sequence into the backbone reference tree. Using these tools, environmental sequences can be placed into the Glomeromycota phylum – describing putative AMF – and further into the major 11 families. Importantly, the concerted effort packaging this process into a formal pipeline improved accessibility of this phylogenetic approach for interested scientists.

Despite the benefit to the research community brought by the new pipeline, users unfamiliar with command line interfaces and with low computing cluster support struggled with implementation, resulting in a substantial hurdle to the systematic adoption of this pipeline for those using the LSU. The main issues with implementation are trouble installing packages as required by the pipeline (specific versions and packages that require a conda environment, Anaconda 2024) and cluster specific assumptions in bash job scripts. Here, we update the database and pipeline by (1) expanding the backbone tree to include newly described genera and (2) further improve usability of this pipeline, including creating a conda requirements package hosting all required packages and testing the pipeline across three university clusters. This updated backbone reference tree and user-friendly pipeline will contribute to broadened adoption of this tool, ultimately improving the scientific community’s understanding of the ecology of these important fungi (Table 1).

**Table 1:** Improvements to the pipeline

Major improvement	Explanation
1. Backbone tree updated	Four new genera were added to the tree to reflect taxonomic discoveries.
2. Condensed ReadMe	The ReadMe has been condensed to concisely, but clearly, outline steps in the pipeline.
3. Simplified conda installation	A conda requirements file is installed once and includes all required programs and R packages in specified versions.
4. Cross computing cluster compatibility	Several changes have been made to the scripts to be compatible with the three SLURM clusters environments tested.
5. Public repository tracking changes	Our Github repository has all implemented changes tracked; we will continue to update any changes here.

## Expanded backbone reference tree

**Fig. 1:**
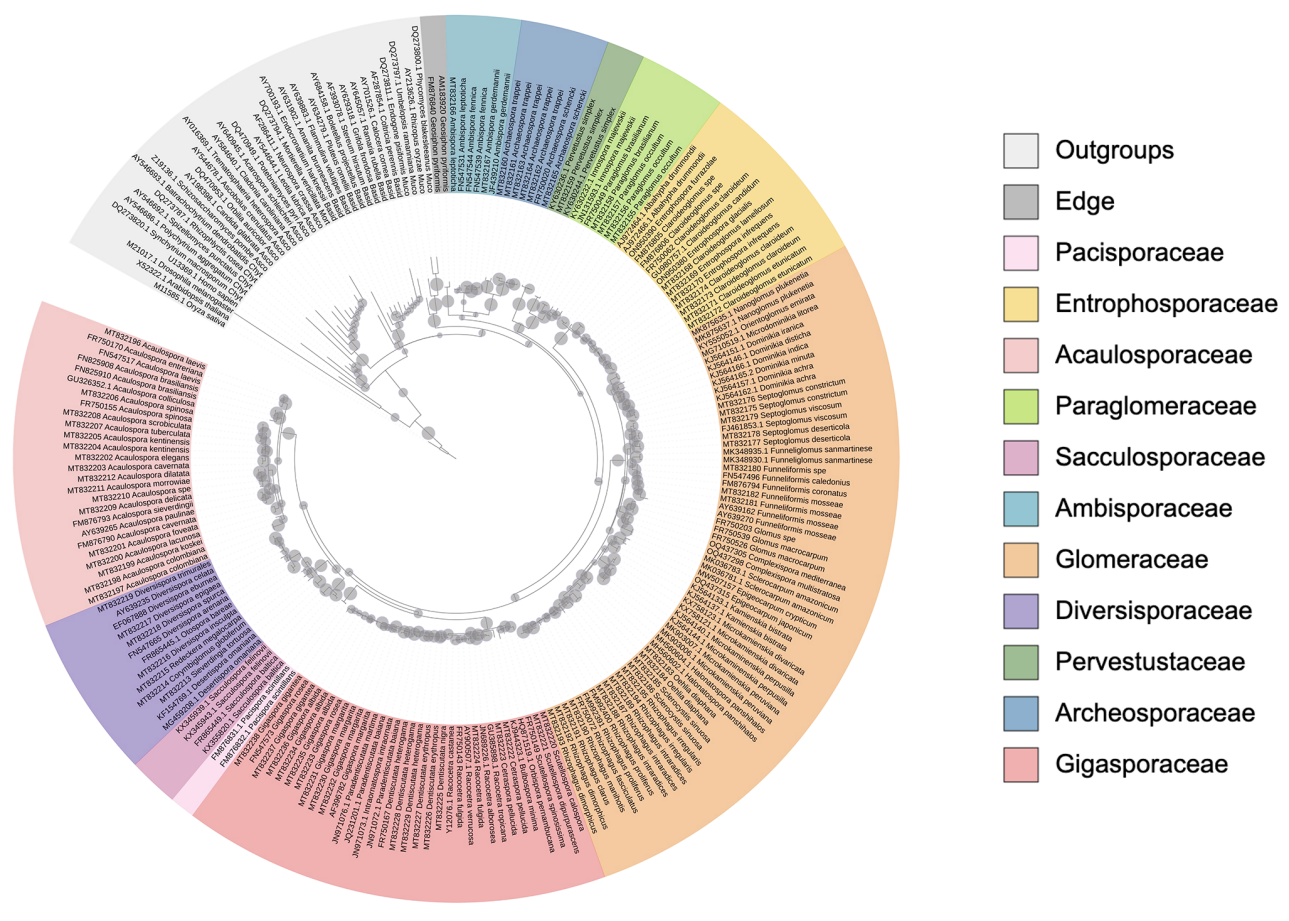
Updated AMF LSU backbone tree

We expanded the backbone reference tree used in the pipeline (Delavaux et al. 2021; Delavaux et al. 2022) to include several new genera and species, reflecting changes occurring since the reference tree was first created (Fig. 1, version 16). In particular, we add species from the four genera *Epigeocarpum*, *Silvaspora*, *Complexispora*, and *Entrophospora* (Błaszkowski et al. 2021a, b, 2022, 2023; da Silva et al. 2023; NCBI accession MW507157.1, MW541060, OQ437298, OQ437305, OQ437315.1, ON950380.1, ON950390.1). We used the start of the LSU region using primers LROR/FLR2 (Bunyard et al. 1994; Trouvelot et al. 1999; ~700–900 bp) and built our backbone tree using RAxML 8.2.12 (Stamatakis 2014) with 1000 bootstrap replicates and the evolutionary model GTRGAMMA. In parallel, we have updated all pipeline scripts to reflect this novel tree. Future work could expand the current backbone tree further by allowing for phylogenetic extraction of genera and even species. Further improvements will be integrated into our Github repository (https://github.com/c383d893/AMF-LSU-Database-and-Pipeline2) as new taxa are described.

## One installation for the entire pipeline

Although previous formalizing of the database and pipeline helped improve accessibility to phylogenetic placement for the LSU region, even this streamlined version may be intimidating to those who are unfamiliar with SLURM (Simple Linux Utility for Research Management) cluster environments and the linux command line. Therefore, here, we provide a new simple installation mechanism (conda requirements file, https://github.com/conda/conda) that requires only one installation per user. This substantially reduces the installation burden as compared to the previous pipeline, which required five program and six R package installations. To allow for this single installation containing all required programs and packages, we begin from a stable base of qiime2 2024.2n (Bolyen et al. 2019) to which we add a single R package (treetools; Smith 2019). Not only does this approach require a single installation, but the required programs and packages remain unchanged, with versions pre-set. This reduces any unforeseen version incompatibility within the pipeline. In addition, we also replaced a dependency external to qiime2 (fastqc; Andrews 2010) for read quality visualization. Instead of relying on fastqc, we now use qiime2’s built in visualization commands paired with qiime’s online visualization tool (view.qiime2.org). Put simply, this approach allows users to install one ‘meta package’ or folder (the conda environment) containing all packages in the exact versions required for the pipeline to run; when running the pipeline, the user simply loads this environment and can be confident that all packages and commands are exactly as intended. This new single installation will not only make it easier for all users, but especially allow for less experienced users with low cluster support to access and benefit from the pipeline.

## Ground truthing ease of implementation across three computing clusters

Previous development and testing of the pipeline was conducted on only one computing cluster at the University of Kansas, raising concerns of transferability of the pipeline across different SLURM environments. Importantly, small differences in cluster setup can lead to script failure, presenting an unnecessary difficulty for those implementing the pipeline for their own research. Therefore, this current pipeline was tested and adapted to work consistently across three different university computing clusters. Specifically, we have tested the pipeline using ten samples of data from tallgrass prairie soil samples on the (1) University of Kansas, (2) University of Colorado Boulder (Alpine), and (3) ETH Zurich (Euler) computing clusters. The ETH Zurich cluster does not support or install conda environments for users at this time, making it the ideal test for the newly integrated conda environment. The successful implementation of the pipeline on a cluster with no conda support gives us confidence that it should function smoothly at other clusters. We retain the ability to choose ASV or OTU outputs – via two versions of the pipeline – and ensure both pipelines have consistent naming and updates. Another change we implemented was explicitly directing temporary files, as this was causing issues on certain clusters (due to cluster level default differences). All temporary files should now consistently write to the main project folder, and be removed upon completion, independent of cluster configuration. Together, this testing across two continents and three university computing clusters increases transferability and use of the pipeline.

## Conclusions

We present an updated user-friendly database and pipeline for environmental placement of arbuscular mycorrhizal fungi using the LSU rDNA gene region. Our major improvements include (1) providing an expanded backbone reference tree to reflect recently described taxa (2) streamlining the requirements for users to implement the pipeline on a cluster relying on the most common workload manager (SLURM) and (3) minimizing likelihood of cluster specific issues by testing the pipeline with the same dataset across three computing clusters. These improvements may further increase the utility of the LSU region for identification of AMF and AMF families of known and previously undescribed AMF.

### Electronic supplementary material

Below is the link to the electronic supplementary material.

Supplementary Material 1

## Data Availability

All sequencing data has been uploaded to NCBI BioProject PRJNA1106240. All code for the AMF LSU pipeline is available at Github repository https://github.com/c383d893/AMF-LSU-Database-and-Pipeline2.
