# Supplementary figures and images for: An updated LSU database and pipeline for environmental DNA identification of arbuscular mycorrhizal fungi
Source: Mycorrhiza. 2024 Jun 29;34(4):369–73. doi: 10.1007/s00572-024-01159-3 (PMC11283431; doi:10.1007/s00572-024-01159-3)

Figure S1 | Updated AMF LSU backbone tree with bootstrap support


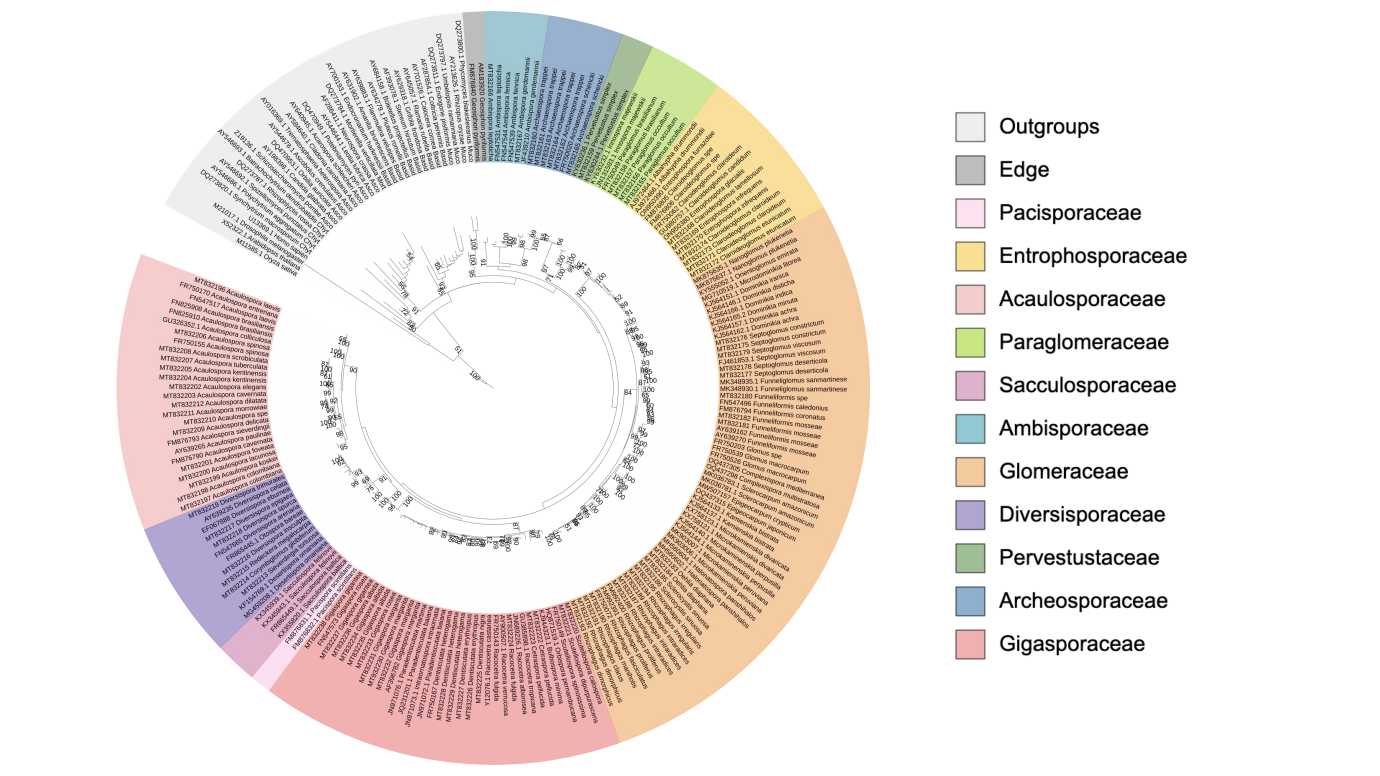

Supplement: Supplementary file 1 — Supplementary Material 1 [file 572_2024_1159_MOESM1_ESM.docx]
